# Pose Estimation of Omnidirectional Camera with Improved EPnP Algorithm

**DOI:** 10.3390/s21124008

**Published:** 2021-06-10

**Authors:** Xuanrui Gong, Yaowen Lv, Xiping Xu, Yuxuan Wang, Mengdi Li

**Affiliations:** College of Optoelectronic Engineering, Changchun University of Science and Technology, Changchun 130022, China; 2019200040@mails.cust.edu.cn (X.G.); xxp@cust.edu.cn (X.X.); 2019100213@mails.cust.edu.cn (Y.W.); 2017100212@mails.cust.edu.cn (M.L.)

**Keywords:** omnidirectional camera, pose estimation, Perspective-n-Point

## Abstract

The omnidirectional camera, having the advantage of broadening the field of view, realizes 360° imaging in the horizontal direction. Due to light reflection from the mirror surface, the collinearity relation is altered and the imaged scene has severe nonlinear distortions. This makes it more difficult to estimate the pose of the omnidirectional camera. To solve this problem, we derive the mapping from omnidirectional camera to traditional camera and propose an omnidirectional camera linear imaging model. Based on the linear imaging model, we improve the EPnP algorithm to calculate the omnidirectional camera pose. To validate the proposed solution, we conducted simulations and physical experiments. Results show that the algorithm has a good performance in resisting noise.

## 1. Introduction

The Perspective-n-Point (PnP) problem is a classic problem in computer vision. The aim is to calculate the orientation of a camera given its intrinsic parameter and a set of correspondences between 3D points and their 2D points. It is widely used in computer vision. In 2008, Xu [1] used an auxiliary point to extend the linear method for the special case of four coplanar points and find the coarse solutions for the general P3P problem. In 2012, Li [2] proposed a solution for the PnP problem that can retrieve the optimum by solving a seventh-order polynomial. In 2018, Wang [3] transferred the problem to solve a seventh-order and a fourth-order univariate polynomial. In 2020, Zhou [4] constructed the PnP problem for an uncalibrated camera as a 20th-order polynomial system. In 2021, Meng [5] mitigated scale bias by multiplying an independent inverse average depth variable onto the object space error to improve the accuracy of pose estimation.

Recently, omnidirectional vision system has become a hot topic for researchers in some fields such as robot driving [6,7], augmented reality [8], and video surveillance [9,10,11]. An omnidirectional vision system provides a 360-degree panorama in the horizontal direction and is composed of a common CCD and mirror. It can help reduce the number of cameras needed and overall costs. For omnidirectional camera pose estimation, the control point coordinates around the camera improve the accuracy of camera pose estimation. A large field of view can effectively reduce the loss of tracking caused by matching. So, the problem of pose estimation based on an omnidirectional vision system is important. In 2001, Daniel G. Aliaga [12], an earlier researcher in this research direction, proposed a pose estimation algorithm based on coded points. He set up a complete stand-alone system, but the method is only applicable for parabolic mirror omnidirectional camera. In the same year, Paulino [13] proposed a method to estimate the pose of a center omnidirectional camera with an arbitrary mirror surface. Gebken [14] presented a novel perspective pose estimation for omnidirectional vision that involves a parabolic central catadioptric sensor by using small data sets, combining geometry and stochastics such that they obtain pose from only three image points. Goncalvep [15] proved that any reflection point belongs to an analytical quadric that intersects the mirror quadric itself and presented a linear method to estimate the pose for the noncentral omnidirectional system. Ilizirov [16] proposed deriving the metric between internal reflections; a closed form similar to the principle of collinearity was obtained and then extended to a linear model. Miraldo [17] derived parametric equations to estimate pose for vanishing points and vanishing curves by using the calibration parameters and mirror shape coefficients. Due to the nonlinear imaging of omnidirectional cameras, a high-order constraint or more complicated formula is needed to establish the imaging model or camera pose estimate. In this paper, we establish a relationship between the omnidirectional image and the traditional camera image to simplify the imaging system.

In 2009, Lepetit [18] proposed the EPnP algorithm, which is widely used to estimate the pose of the conventional transmission camera. The EPnP algorithm speeds up the calculation by reducing O(3) to O(1) and introduces four reference control points to reduce the influence of a single control point error. Since the EPnP algorithm was proposed, it has received widespread attention, and some researchers have also proposed algorithms derived from the EPnP algorithm [19,20,21]. Based on EPnP algorithm, Penatesanchez [19] proposed a pose estimation algorithm with unknown camera focal length. In the same year, Deng [20] applied the EPnP algorithm to mosaic images. In 2018, Chen Peng [21] improved the EPnP algorithm by the Gauss-Newton method to optimize the coordinates of virtual control points in the camera coordinate system.

However, in the EPnP algorithm, linear equations are established based on imaging systems to solve the camera pose. This paper analyzes the imaging model of the omnidirectional camera and presents the linear virtual imaging plane. We derive the linear imaging equation of the omnidirectional camera and put forward a method that estimates omnidirectional camera pose by the EPnP algorithm. First of all, the control points of an omnidirectional image are projected into a linear virtual image plane. Then, the control point location in the virtual camera coordinate system, solved by the EPnP algorithm, is converted into the location of the omnidirectional camera coordinate system. Finally, the omnidirectional camera pose is solved by an absolute orientation method. Section 2 describes the spherical model of an omnidirectional camera. Section 3 describes the specific algorithm. Section 4 and Section 5 respectively describe simulations and real image experiments and verify the noise immunity and reliability of this method.

## 2. Omnidirectional Camera Spherical Model

The omnidirectional vision system is composed of a camera and a mirror. Mei presented an omnidirectional camera spherical model and corresponding calibration toolbox [22]. They proved the equivalence of the reflection process and spherical mapping.

First, the spatial point **X^c^** with coordinate (**X**^c^, **Y**^c^, **Z**^c^) is projected onto the unit sphere to obtain point **X^m^**.

Then, in the new reference frame centered with (0, 0, −*ξ*) as the origin, **X^m^** is changed to **X^s^**.

Next, the point **X^s^** is projected onto a normalized plane to obtain the point **u^s^**.

The last projection is related to a generalized camera projection matrix **K**. The point **u^s^** on the normalized plane is projected onto the image plane by matrix **K**.

**Figure 1:**
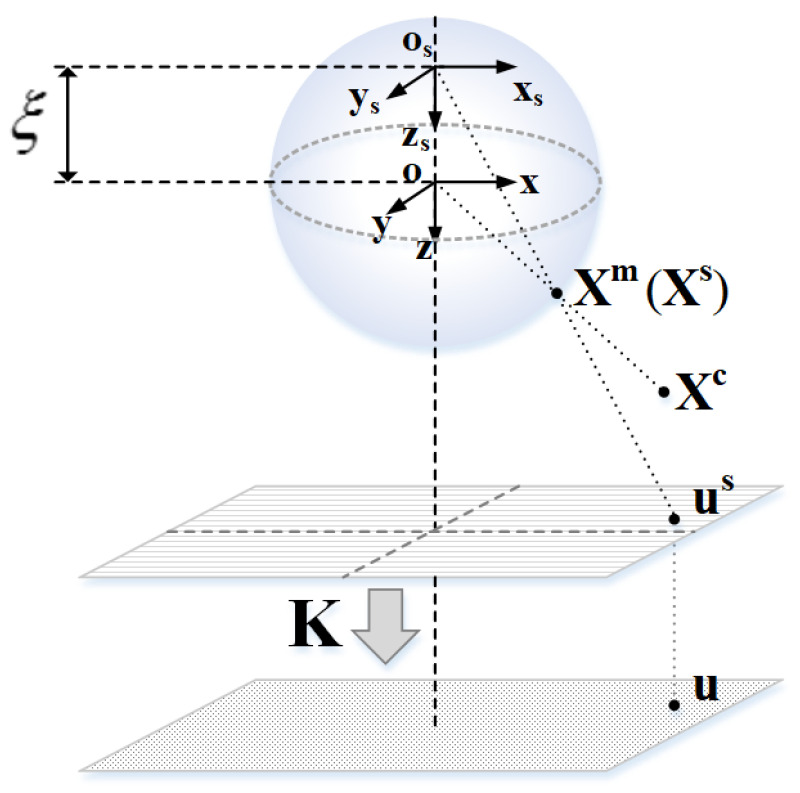
Unit spherical model.

As shown in Figure 1, the relationship between an arbitrary 3D space point **X^c^** and corresponding image plane point **u** can be written as:

(1) $$\lambda u_{i}=K\cdot h\left(\frac{X_{i}^{c}}{\left\|X_{i}^{c}\right\|}\right)$$

with $K=\left[\begin{array}{ccc} f_{1}\eta & f_{1}\eta \alpha & u_{0}\\ 0 & f_{2}\eta & v_{0}\\ 0 & 0 & 1 \end{array}\right]$, $h\left(X_{i}^{{}^{m}}\right)=\left[\begin{array}{c} \frac{X_{i}{}^{m}}{Z_{i}^{m}+\xi }\\ \frac{Y_{i}^{m}}{Z_{i}^{m}+\xi }\\ 1 \end{array}\right]$.

where $\left\|X_{i}^{c}\right\|$ is norm of $X_{i}^{c}$. The function h represents an expression of a spatial point to a normalized plane. *ξ* and *η* are related to the mirror of the omnidirectional camera. Table 1 shows the values of *ξ* and *η* in different mirrors. *f*_1_ and *f*_2_ are the focal lengths of the camera in the X and Y directions, respectively. α is the correlation coefficient between the X and Y directions and is usually equal to zero. A generalized camera projection matrix indicates we are no longer considering the sensor as a separate camera and mirror but as a global device. *f* and *η* cannot be estimated independently. We will note *f*_x_ = *f*_1_*η* and *f*_y_ = *f*_2_*η*.

The camera calibration process can obtain the projection matrix **K** and the coefficients *ξ*.

## 3. Pose Estimation Algorithm

The traditional camera pinhole model, camera optical center, image point, and space control point meet the collinear condition. According to the characteristic, we introduce virtual imaging that is the process of projecting virtual space point coordinates **X^vir^** onto the virtual plane **u^vir^**. The logic of the approach is shown in Algorithm 1. We respectively calculate the coordinate transformation from image point to virtual image point and from space point to virtual space point.
**Algorithm 1.** Logic of omnidirectional camera pose estimation algorithms.1. **Data acquisition.** Extract corner point **u** and obtain its coordinate in the world coordinate system **X^w^**2. **Determine camera.** Determine the number and location of virtual cameras3. **Coordinate transformation.** Convert corner coordinate to virtual image plane coordinate.4. **Calculate X^vir^.** Use modified EPNP algorithm to calculate virtual camera coordinate **X^vir^**.5. **Coordinate transformation.** Convert virtual camera coordinate to camera coordinate.6. **Calculate pose.** Calculate the camera pose according to **X^c^** and **X^w^**

**Figure 2:**
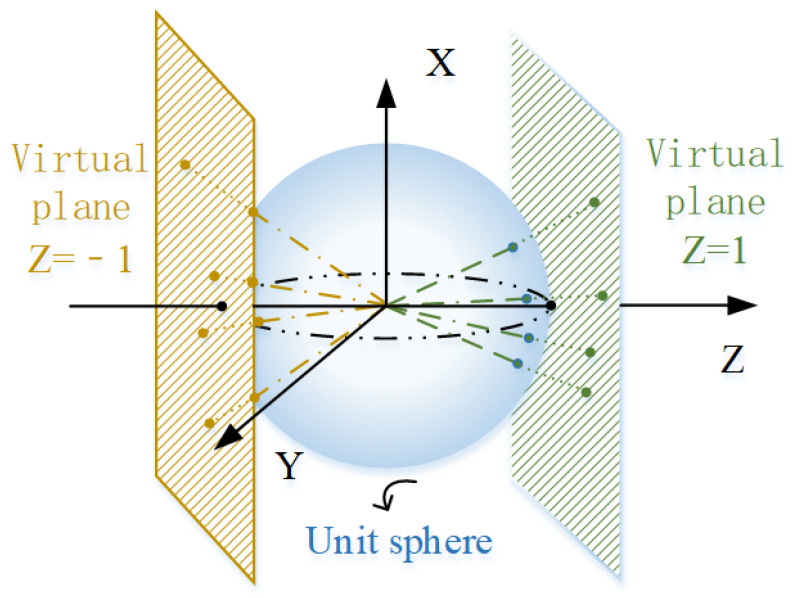
Virtual camera coordinate system.

As shown in Figure 2, two virtual cameras are established to substitute the omnidirectional camera. The optical center of the virtual camera is coinciding with the optical center of the omnidirectional camera, in which optical axes are aligned with the positive and the negative directions of the *Z*-axis. In a virtual camera, the conversion from the image coordinate system to the pixel coordinate system is not important, so we simplify this part. The virtual image plane is actually the normalized plane in the perforation imaging model, that is, the Z=1 or Z=−1 plane.

After determining the number of virtual cameras and the position of the virtual image plane, the next step is to calculate the coordinates of the virtual points. First, the image point **u** is projected onto the unit sphere. Through algebraic operations, we can obtain an equation from the image point to the corresponding unit sphere.

(2) $$X^{m}=h^{-1}(K^{-1}u)$$

(3) $$h^{-1}\left(u^{s}\right)=\left[\begin{array}{c} \frac{\xi +\sqrt{1+\left(1+\xi^{2}\right)\left(u^{s}{}_{i}^{2}+v^{s}{}_{i}^{2}\right)}}{u^{s}{}_{i}^{2}+v^{s}{}_{i}^{2}+1}\cdot u^{s}{}_{i}\\ \frac{\xi +\sqrt{1+\left(1+\xi^{2}\right)\left(u^{s}{}_{i}^{2}+v^{s}{}_{i}^{2}\right)}}{u^{s}{}_{i}^{2}+v^{s}{}_{i}^{2}+1}\cdot v^{s}{}_{i}\\ \frac{\xi +\sqrt{1+\left(1+\xi^{2}\right)\left(u^{s}{}_{i}^{2}+v^{s}{}_{i}^{2}\right)}}{u^{s}{}_{i}^{2}+v^{s}{}_{i}^{2}+1}-\xi \end{array}\right]$$

where (*u***^s^***, v^s^*) are the coordinates of **u^s^′**. *h*^−1^ is essentially the inverse of *h* in Equation (1). The coordinate of $X^{m}$ satisfies the equation $X^{m}{}^{2}+Y^{m2}+Z^{m2}=1$.

Then, we normalize the *Z^m^* coordinate to obtain the virtual image plane coordinates.

(4) $$\left[\begin{array}{c} u_{i}{}^{vir}\\ v_{i}{}^{vir}\\ 1 \end{array}\right]=\frac{1}{Z_{i}{}^{m}}\left[\begin{array}{c} X_{i}{}^{m}\\ Y_{i}{}^{m}\\ Z_{i}{}^{m} \end{array}\right]\mathrm{or}\left[\begin{array}{c} u_{i}{}^{vir}\\ v_{i}{}^{vir}\\ -1 \end{array}\right]=\frac{1}{-Z_{i}{}^{m}}\left[\begin{array}{c} X_{i}{}^{m}\\ Y_{i}{}^{m}\\ Z_{i}{}^{m} \end{array}\right]$$

While the virtual image *Z* is equal to 1, the virtual camera coordinate system will coincide with the omnidirectional camera system. If the virtual image *Z* is equal to −1, there is a corresponding relationship between the virtual camera coordinate system and the omnidirectional camera coordinate system: *Z^vir^* = −*Z^c^*, *Y^vir^* = −*X^c^*, and *X^vir^* = −*Y^c^.* The virtual camera imaging equation can be written as:

(5) $$\lambda^{vir}\left[\begin{array}{c} u_{i}^{vir}\\ v_{i}^{vir}\\ 1 \end{array}\right]=K^{vir}X_{i}^{vir}=IX_{i}^{vir}$$

where the $\lambda^{vir}$ is scalar depth parameters. $K^{vir}$ is the internal calibration matrix of the virtual camera. For the distance from the optical center to the image plane, $f^{vir}=1$, and for the principal point of the virtual camera, (*u*_0_, *v*_0_) = (0, 0). So, the virtual camera internal calibration matrix $K^{vir}$ is equivalent to **I**.

In the next step, the virtual image points and imaging equations are brought into the EPNP algorithm to calculate the point coordinates in the virtual camera coordinate system. Suppose the control points in the world coordinate system are {$X_{1}^{w}$ $X_{2}^{w}$ $X_{3}^{w}$ $X_{4}^{w}$ … $X_{n}^{w}$}. $X_{i}^{w}$ (*i =* 1 *… n*) can be written by four reference points $\left(C_{1}^{w},C_{2}^{w},C_{3}^{w},C_{4}^{w}\right)$ in the world coordinate system.

(6) $$X_{i}^{w}=\sum_{j=1}^{4}\alpha_{ij}C_{j}^{w}$$

Because of the nature of Euclidean space, the same relationship holds in the virtual camera coordinate system. We define the reference points in the virtual coordinate system as $C_{1}^{vir},C_{2}^{vir},C_{3}^{vir},C_{4}^{vir}$. The following equation can be written:

(7) $$X_{i}^{vir}=\sum_{j=1}^{4}\alpha_{ij}C_{j}^{vir}$$

We expand Equation (5) by Equation (7) and 3D coordinates of each reference point.

(8) $$\lambda \left[\begin{array}{c} u_{i}^{vir}\\ v_{i}^{vir}\\ 1 \end{array}\right]=\left[\begin{array}{ccc} 1 & 0 & 0\\ 0 & 1 & 0\\ 0 & 0 & 1 \end{array}\right]\cdot \sum_{j=1}^{4}\alpha_{ij}\left[\begin{array}{c} x_{j}^{vir}\\ y_{j}^{vir}\\ z_{j}^{vir} \end{array}\right]$$

Substituting in the first and two rows, two linear equations can be obtained:

(9) $$\{\begin{array}{c} \sum_{j=1}^{4}\alpha_{ij}x_{j}^{vir}-\alpha_{ij}u_{i}^{vir}z_{j}^{vir}=0\\ \sum_{j=1}^{4}\alpha_{ij}y_{j}^{vir}-\alpha_{ij}v_{i}^{vir}z_{j}^{vir}=0 \end{array}$$

The unknown parameters of the expression are only related to reference point coordinates. Rewrite Equation (10) to matrix form:

(10) Μx=0

where **M** is a 2n × 12 matrix, and $x={\left[\begin{array}{cccc} C_{1}^{vir}{}^{T} & C_{2}^{vir}{}^{T} & C_{3}^{vir}{}^{T} & C_{4}^{vir}{}^{T} \end{array}\right]}^{T}$ is a vector. Vector **x** belongs to the right null space of **M** matrix. **x** is solved by singular value decomposition.

(11) $$x=\sum_{k=1}^{N}\beta_{k}v_{k}$$

**v_k_** is an eigenvector with zero eigenvalues of **M**, with scalar coefficients. If *N* is determined, reference point coordinates can be calculated by Equation (11). We can obtain **X*^vir^*** in different virtual camera coordinates system by Equation (7). Then, we reproject **X*^vir^*** to the omnidirectional camera system to obtain the location $X_{i}^{c}\;$.

At last, the absolute orientation algorithm [23] is used to directly calculate the rotation matrix **R** and translation vector **T** of the omnidirectional camera coordinate system relative to the object space coordinate system, that is, the pose of the omnidirectional camera.

(12) $$X_{i}^{w}=RX_{i}^{c}+T\;$$

with $A=\sum_{i=1}^{n}X_{i}^{wo}{\left(X_{i}^{co}\right)}^{T}$.

The relationship between $X_{i}^{wo}$ and $X_{i}^{w}$ is shown as Equation (13), and the relationship between $X_{i}^{co}$ and $X_{i}^{c}$ is shown in Equation (14).

(13) $$X_{i}^{wo}=X_{i}^{w}-\frac{1}{n}\sum_{i=1}^{n}X_{i}^{w}$$

(14) $$X_{i}^{co}=X_{i}^{c}-\frac{1}{n}\sum_{i=1}^{n}X_{i}^{c}$$

## 4. Results

### 4.1. Simulation

We produced synthetic 3D–2D correspondence by a virtual calibrated camera, the intrinsic parameters of which are shown in Table 2. Calibration parameters were selected empirically. Rotation matrix and translation vector were randomly generated. The translation vector norm was distributed into [100, 200]. In the experiment, we generated 10 sets for the input data and kept the number of control points for each setting unchanged.

#### 4.1.1. Synthetic Experiments of Accuracy about Noise

**Figure 3:**
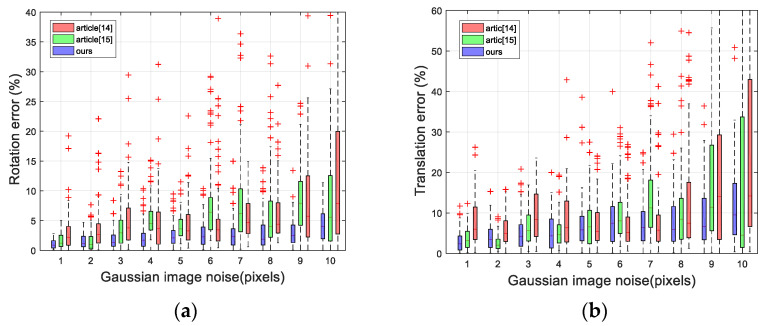
Pose estimation errors for different noise. (**a**) Rotation matrix error (**b**) Translation vector error.

In the first experiment, we assessed the effect of the coordinate error on the accuracy of pose estimation. The Gaussian noise, having zero mean and standard deviation from 0 to 10, was added to the corresponding 2D point coordinates. For each level of standard deviation, we performed 100 experiments. The results of the experiment are shown in Figure 3a,b.

We computed the relative error of the estimated rotation by Equations (15) and (16), where q and $q_{ture}$ are the normalized quaternion corresponding to the rotation matrix.

(15) $$E_{rot}(\%)=\left\|q_{ture}-q\right\|$$

(16) $$E_{trans}(\%)=\frac{\left\|t_{ture}-t\right\|}{\left\|t\right\|}$$

In Figure 3a,b, in addition to the rotation error, we also plotted the translation error. The line in the box is the average error. The rectangle represents 50% error distribution. The smaller it is, the more concentrated the error distribution, indicating that the method is more stable. It can be observed that the error of our method grows linearly with the level of noise and remains much lower than all the others. When the noise level is small, the accuracy of the algorithms of [14,15] is similar to that of the algorithm in this paper. When the noise level is large, the error of these two algorithms is large. When the variance of Gaussian noise is 10, the maximum error of the rotation angle is below 10%, the average error of the rotation angle is 3%, and the average error of the translation vector is 12%, which indicates that it has good antinoise performance.

#### 4.1.2. Synthetic Experiments of Accuracy about the Number of Control Points

**Figure 4:**
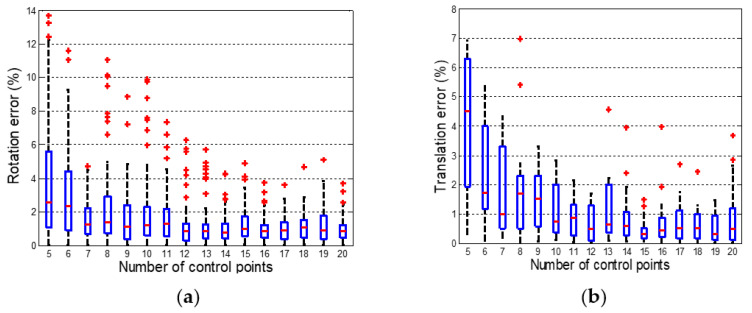
Pose estimation errors for different numbers of control points. (**a**) Rotation matrix error (**b**) Translation vector error.

Simulation experiments were conducted to explore the influence of the number of control points on the pose estimation accuracy. The number of control points on a single surface was varied from 5 to 20, keeping the average noise value at 0 and the standard deviation at 2. The rotation matrix and translation vector were randomly generated, where the norm of the translation vector was [100, 200]. One hundred experiments were performed, and the results are shown in Figure 4a,b.

The error decreases as the number of control points increases. Figure 4 shows that the error of the pose estimation is related to the number of control points projected on the virtual planes (plane *Z =* −1 and plane *Z =* 1). When the number of control points is greater than 12, the accuracy approaches stability. When the number of points is less than 6, the pose estimation error increases. For the case where the number of control points is between 6 and 12, the error is uncertain. If the number of control points on each virtual plane does not exceed 6, the error is large. If there are more than 6 control points on a virtual plane, the accuracy is better. The 4 spatial 3D control points have 12 unknown parameters. As shown in Equation (10), two equations are obtained for each control point. Therefore, when the number of control points is greater than or equal to 6, the system has a definite solution.

### 4.2. Real Images

#### 4.2.1. Calculating Rotation Angle

**Figure 5:**
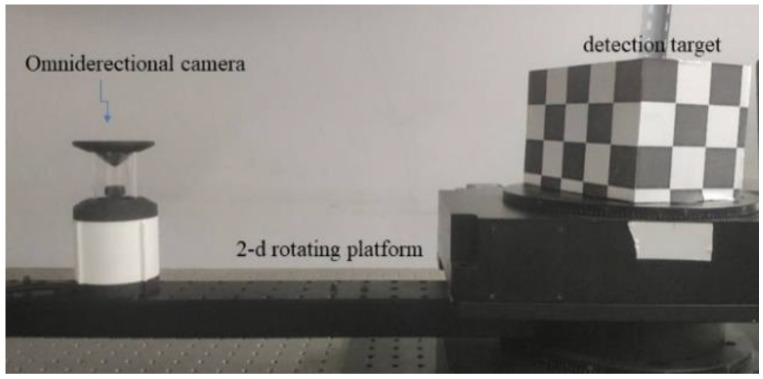
Experimental layout.

In the first experiment with real images, we applied the rotating platform to survey the motion matrix of the detection target. The object in the picture was a checkerboard pattern of 10 cm × 10 cm × 20 cm carved with 30 mm × 30 mm. The experimental layout is shown in Figure 5. Assuming that ϕ is the rotation angles on the axes of rotation, the rotation matrix **R** can be easily obtained by the Rodriguez formula.

We randomly changed the angles *ϕ* and then captured images. We ignored the difference between the center of the turntable and the origin of the world coordinate system. The coordinates of the checkerboard corners were obtained with the implementation of key point recognition by the method of [24]. This is a gradient-based subpixel intersection detection algorithm. The internal parameter was obtained by running the camera calibration toolbox [22]. The calibration results are shown in Table 3. Then, the pose was estimated by the proposed method and the methods in [14,15].

The pose of the calibration target from camera coordinate system to world coordinate system was calculated before and after exercise, expressed by **R_1_**, **R_2_** and **T_1_**, **T_2_**, respectively. The conversion from the world coordinate system before movement to the world coordinate system after movement is as follows:

(17) $$\begin{array}{c} X_{w1}=R_{1}{}^{-1}R_{2}X_{w2}-R_{1}{}^{-1}[T_{2}-T_{1}]\\ =R_{w1-w2}X_{w2}+T_{w1-w2} \end{array}$$

**Figure 6:**
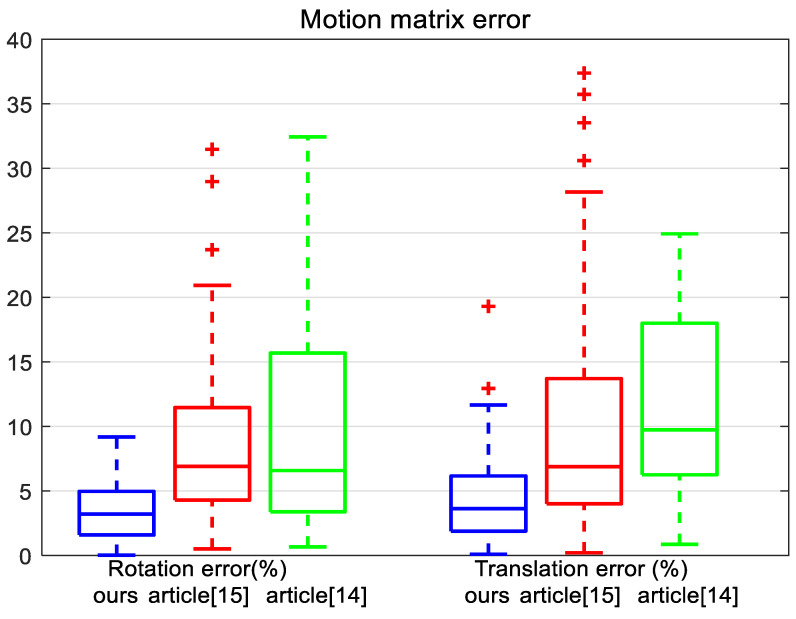
Comparing the accuracy of our approach method, the method of [14], and the method of [15].

The measured rotation matrix was converted into a form of quaternions and then compared with the measured result. Figure 6 shows calculated the rotation errors and translation errors when the camera position was fixed at 30 mm of the caliper and the rotation angle of the turntable was varied.

In the result of the algorithm of [15], the error distribution is relatively scattered. The line segment representing the average value is in the lower middle position, indicating that most of the most data have small errors and a small part of data have large errors. In the result of the algorithm of [14], there are some large discrete values above the box. This is the fault tolerance of pose calculation due to the plane incline. The results of the proposed algorithm are relatively concentrated. The line segment representing the average value is in the middle of the whole box, which indicates that the distribution of error values is relatively average and the algorithm has good stability. The average value rotation error is about 2.5% and the average value translation error is about 3.0%. Compared with the results of the simulation experiment, both the rotation error and the translation error of the real image experiment become larger. The reason is that the measurement error of camera calibration is more complicated than the simulation error. Rotation angle is calculated from two pictures before and after, which requires higher stability of the algorithm. The error of any picture will affect the calculation of rotation angle.

#### 4.2.2. Reconstruction

**Figure 7:**
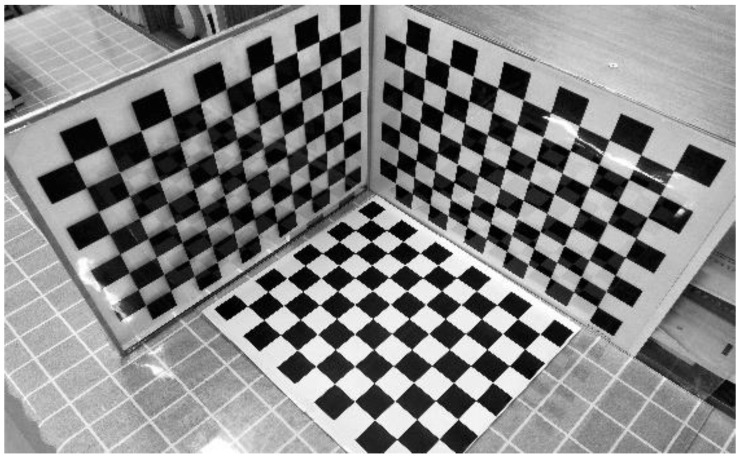
The sample trihedron used for the 3D reconstruction experiment.

In the second experiment, we reconstructed the 3D metric of the object by using two omnidirectional images. In order to study the accuracy of the reconstruction, the object consisted of three orthogonal checkerboard patterns with the size of each square measuring 30 mm × 30 mm, as shown in Figure 7.

**Figure 8:**
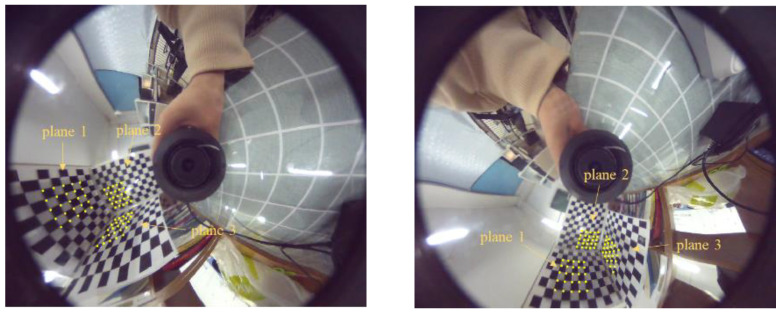
Two pictures of the trihedron taken by the omnidirectional camera. The points used for the 3D reconstruction are indicated by yellow dots.

**Figure 9:**
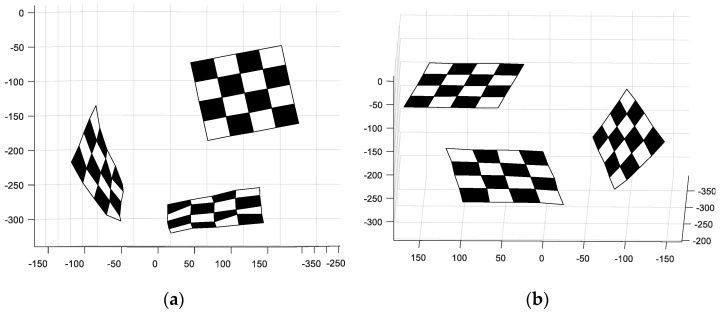
Three rendered views of the reconstructed trihedron. (**a**,**b**) are the results of different perspectives.

We used the omnidirectional camera to take images (see Figure 8) at two different locations. The corner points were manually selected and matched. The pose of the object was estimated by the method proposed in this paper. Then, the relative rotation matrices and translation vectors between the two images were derived. The reconstruction results are shown in Figure 9. In order to compare the reconstructed value with the real value, the least square was used to fit the plane. The angles between the planes were 89.27°, 89.44°, and 89.64°. Finally, we calculated the dimensions of the reconstructed checkerboard. The average error relative to the ideal was 1.85 mm. We also used the methods proposed in [14,15] to conduct experiments. The plane included angles were 95.6°, 85.7°, and 84.5° for the method of [14] and the average error of the corner coordinates was 3.17 mm. The plane included angles were 93.2°, 84.6°, and 83.5° for the method of [15], and the average error of the corner coordinates was 2.65 mm.

The inclination of the plane where the space point is located has a great influence on the pose estimation. When the points are lying on a plane on an ideal plane facing the camera, there is almost nonexistent pose ambiguity, and all the methods have similar accuracy, with almost nonexistent outliers for all the methods. The pose ambiguity problem appears for inclined planes. The reprojection error of the camera pose estimated by the algorithms in [14,15] on plane 1 was small, while the reprojection error on planes 2 and 3 was large.

### 4.3. Discussion

In this paper, by adding a virtual image plane, the nonlinear imaging process of the omnidirectional camera is transformed into an equivalent linear imaging process. The EPnP algorithm is then applied to the omnidirectional imaging system. The control point coordinates around the camera improve the accuracy of camera pose estimation. A large field of view can effectively reduce the loss of tracking caused by matching. The EPnP algorithm converts the control points into four virtual coordinates to reduce the influence of a single control point on pose estimation, and it has good antinoise performance. In addition, the control point modification of this method only contains the basic mapping relation, and the calculation cost is low. Specifically, the execution time of the coordinate map is about 20 milliseconds per frame, and the execution time of pose estimation is about 10 milliseconds. We think there is room for improvement if the code can be executed on the GPU. However, this method has some limitations in the number of virtual image faces to choose. When more virtual image planes are selected, more control points are needed, and fewer image planes are selected, which leads to larger errors caused by camera approximation. Our plan is to remove these limitations in the future through the adoption of deep learning technologies.

## 5. Conclusions

In this work, we propose the linear equivalent model for omnidirectional cameras. The omnidirectional camera is equivalent to the combination of two or more virtual cameras. After solving the nonlinear imaging problem of the omnidirectional camera, the EPnP algorithm was extended to the omnidirectional camera. The method can be suitable for all kinds of mirror omnidirectional systems. In the simulation part, we first studied the influence of image point error on pose estimation. Results show that the proposed solutions work well when noise occurs. Then, we investigated the influence of the number of control points on the accuracy of pose estimation, and the accuracy was found to increase with the increase in the number of control points. The four virtual control points in the EPnP algorithm effectively reduce the influence of a single spatial control point on the overall pose estimation. The better antinoise performance of the method was substantiated by simulation and real image experiments. At the same time, space points around the camera can effectively improve the positioning accuracy, which is an advantage brought by the large field of view. In the second experiment of the real image, we put the pose estimation results into the reconstruction algorithm. Three checkerboard calibration plates were reconstructed. We calculated the included angle of the checkerboard calibration plate to evaluate the reconstruction accuracy. In future research, we will extend the EPnP algorithm to the scene reconstruction of the panoramic camera.

In addition, the virtual image plane can solve the problem of large image distortion by retaining the large field of view. The imaging process of the virtual camera is linear. This can speed up the popularization of omnidirectional cameras in a wide range of video surveillance, robot navigation, and other applications in computer vision.

This work has been focused on omnidirectional camera pose estimation. Future work will also be dedicated to location and reconstruction with a large field of view of the system. The powerful tools of deep learning (DL) will also be taken into account for camera positioning by directly processing large field images.

## Figures and Tables

**Table 1:** Unified model parameters.

Parameter	ξ	η
Parabola	1	−2p
Hyperbola	$\frac{d}{\sqrt{d^{2}+4p^{2}}}$	$\frac{-2p}{\sqrt{d^{2}+4p^{2}}}$
Ellipse	$\frac{d}{\sqrt{d^{2}+4p^{2}}}$	$\frac{2p}{\sqrt{d^{2}+4p^{2}}}$
Planar	0	−1

**Table 2:** Internal parameters of the camera in the simulation experiment.

Parameter	*f*_x_	*f*_y_	u_0_	v_0_	ξ
Value	260.1	259.6	517.1	385.8	0.97

**Table 3:** Calibration result.

Parameter	*f*_x_	*f*_y_	u_0_	v_0_	ξ
Value	370.647	370.018	807.551	597.126	1.027
